# Nuclear actin filaments recruit cofilin and actin-related protein 3, and their formation is connected with a mitotic block

**DOI:** 10.1007/s00418-014-1243-9

**Published:** 2014-07-08

**Authors:** Alžběta Kalendová, Ilona Kalasová, Shota Yamazaki, Lívia Uličná, Masahiko Harata, Pavel Hozák

**Affiliations:** 1 Department of Biology of the Cell Nucleus, Institute of Molecular Genetics of the Academy of Sciences of the Czech Republic, v.v.i., Vídeňská 1083, 142 20 Prague, Czech Republic; 2 Laboratory of Molecular Biology, Graduate School of Agricultural Science, Tohoku University, Tsutsumidori-Amamiyamachi 1-1, Aoba-ku, Sendai, 981-8555 Japan

**Keywords:** Nuclear actin, Transcription, Mitosis, Actin-related protein 3, Cofilin

## Abstract

Although actin monomers polymerize into filaments in the cytoplasm, the form of actin in the nucleus remains elusive. We searched for the form and function of β-actin fused to nuclear localization signal and to enhanced yellow fluorescent protein (EN-actin). Our results reveal that EN-actin is either dispersed in the nucleoplasm (homogenous EN-actin) or forms bundled filaments in the nucleus (EN-actin filaments). Formation of such filaments was not connected with increased EN-actin levels. Among numerous actin-binding proteins tested, only cofilin is recruited to the EN-actin filaments. Overexpression of EN-actin causes increase in the nuclear levels of actin-related protein 3 (Arp3). Although Arp3, a member of actin nucleation complex Arp2/3, is responsible for EN-actin filament nucleation and bundling, the way cofilin affects nuclear EN-actin filaments dynamics is not clear. While cells with homogenous EN-actin maintained unaffected mitosis during which EN-actin re-localizes to the plasma membrane, generation of nuclear EN-actin filaments severely decreases cell proliferation and interferes with mitotic progress. The introduction of EN-actin manifests in two mitotic-inborn defects—formation of binucleic cells and generation of micronuclei—suggesting that cells suffer aberrant cytokinesis and/or impaired chromosomal segregation. In interphase, nuclear EN-actin filaments passed through chromatin region, but do not co-localize with either chromatin remodeling complexes or RNA polymerases I and II. Surprisingly presence of EN-actin filaments was connected with increase in the overall transcription levels in the S-phase by yet unknown mechanism. Taken together, EN-actin can form filaments in the nucleus which affect important cellular processes such as transcription and mitosis.

## Introduction

Actin is a highly abundant intracellular protein essential for maintenance of many cellular functions. It is widely expressed across the species and present in all eukaryotic cell types. In the cytoplasm, actin is present in the form of monomers (globular actin, G-actin), which can polymerize to form filaments (F-actin) that can be specifically visualized by phalloidin. The formation of F-actin is driven by the availability of G-actin subunits—a filament grows when G-actin levels exceed the critical concentration required for polymerization, and a filament shrinks if the critical concentration was not reached. Actin filaments are highly dynamic structures that can assemble or disassemble rapidly based on cell needs.

There are many actin-binding proteins available in the cytoplasm. Depending on their relative binding affinities, they can promote, block or alter the formation of actin filaments. In addition, various actin-binding proteins cross-link actin filaments to form bundles or networks (reviewed in Winder and Ayscough 2005). Such structures are important for the maintenance of cell shape, polarity, mechanical resistance, adhesion and movement.

Actin shuttles between cytoplasm and nucleus employing importin 9 and exportin 6 (Dopie et al. 2012). In the nucleus, actin is present in the form of monomers (Jockusch et al. 2006; Kukalev et al. 2005; McDonald et al. 2006; Obrdlik et al. 2008; Pendleton et al. 2003), yet its ability to form nuclear filaments has been questioned for a long time due to the lack of nuclear phalloidin staining. Eventually, several conditions leading to the formation of nuclear actin polymers have been described. Under various stress conditions (e.g., heat shock, DMSO treatment, virus infection etc.), nuclear actin rods and paracrystals were observed in numerous cell types (reviewed in Hofmann 2009). Moreover, a recent study revealed the presence of actin filaments in nuclei of NIH3T3 cells after overexpression of LifeAct, an F-actin marker, fused to nuclear localization signal (NLS). These filaments were formed after serum induction in a formin-dependent manner (Baarlink et al. 2013). Accumulation and subsequent polymerization of the overexpressed actin in the nucleus was also reported after the disruption of the actin export (Dopie et al. 2012; Stuven et al. 2003). Additionally, Miyamoto et al. (2011) detected actin filaments in nuclei of somatic cells transplanted into oocytes of *Xenopus leavis* using an actin-binding domain of utrophin fused to NLS. Interestingly, the same probe revealed the presence of punctate structures in the nuclei of U2OS cells under physiological conditions which were moreover susceptible to phalloidin staining (Belin et al. 2013). Even though these polymeric structures do not co-localize with any actin-binding proteins, they are found predominantly in the interchromatin space and probably serve as a structural platform that facilitates nuclear organization (Belin et al. 2013).

Even though the state of nuclear actin is not entirely clear, its functional importance has been known for some time. Actin is together with the actin-related proteins required for chromatin remodeling (Ikura et al. 2000; Kapoor et al. 2013; Mizuguchi et al. 2004; Shen et al. 2000; Szerlong et al. 2008; Zhao et al. 1998). Actin also associates with all three RNA polymerases (Hofmann et al. 2004; Hu et al. 2004; Philimonenko et al. 2004) and in cooperation with nuclear myosin 1 (NM1) facilitates transcription initiation and recruitment of chromatin modifying complexes during the elongation phase (reviewed in de Lanerolle and Serebryannyy 2011). Furthermore, actin also participates in RNA processing and export by interacting with heterogenous ribonucleoproteins (hnRNPs; Obrdlik et al. 2008; Percipalle et al. 2002).

From the data available, it seems that the state of nuclear actin engaged in chromatin remodeling complexes and in complex with hnRNPs (Kapoor et al. 2013; Obrdlik et al. 2008; Percipalle et al. 2002) is rather monomeric, whereas in transcription both forms seem to be involved (Miyamoto et al. 2011; Obrdlik and Percipalle 2011; Qi et al. 2011; Wu et al. 2006; Ye et al. 2008; Yoo et al. 2007). Similarly, actin in its polymeric form is essential for the movement of genomic loci throughout the nucleus during transcriptional activation (Dundr et al. 2007; Hu et al. 2008). The presence of polymeric actin in the nucleus is also supported by the findings that various proteins known to bind F-actin in the cytoplasm also localize to the nucleus (reviewed in Castano et al. 2010)) and are implicated in nuclear processes such as transcription (Baarlink et al. 2013; Miyamoto et al. 2011; Obrdlik and Percipalle 2011; Wu et al. 2006; Yoo et al. 2007).

Kokai et al. (2014) have previously reported that ectopically expressed β-actin fused to NLS is imported into the nucleus, where it forms filamentous network. Detailed analysis of the network revealed that distinct actin filaments are branched and cross-linked into parallel bundles. The formation of such structures alters the shape of neuronal-like rat PC12 cells and activates serum response factor (SRF)-mediated transcription. In this study, we employed a similar fusion protein, β-actin fused to enhanced yellow fluorescent protein (EYFP) and to NLS (EN-actin), aiming to explore (1) the formation of EN-actin filaments in the nucleus, (2) contribution of actin-binding proteins to the EN-actin filaments formation and dynamics, (3) association of nuclear EN-actin filaments with complexes where endogenous actin is known to localize, and (4) an effect of the nuclear EN-actin filaments formation on cell cycle and transcription in human osteosarcoma cells (U2OS).

## Materials and methods

### Cells and transfections

U2OS, H1299, HEK293 and human skin fibroblasts were cultured in D-MEM supplemented with 10 % FBS in 5 % CO2/air, 37 °C and humidified atmosphere. Cells were transfected with Lipofectamine 2000 (Life Technologies) and TurboFect (Thermo Scientific) according to manufacturer’s protocol. 2 μg of DNA and 5 μl of Lipofectamine or 3 μl of TurboFect was used to transfect 5 × 10^5^ cells. Cells were incubated for 6 to 12 h with a transfection mix and additional 36 h before fixation and imaging. Linear polyethylenimine (PEI), 25 kDa, was purchased from Polysciences. 1 mg/ml stock solution was prepared and pH adjusted to 7. 9 μl of this solution was mixed with 1.5 μg DNA in serum-free media and incubated for 15 min at room temperature. 5 × 10^5^ cells were incubated with transfection mix for 4 h and then grown for 48 h before imaging.

5 μg of exogenous DNA was delivered into 5 × 10^5^ primary mouse skin fibroblasts by nucleofection using Amaxa nucleofector (Lonza), programme C005. Cells were seeded onto coverslips and imaged 48 h after nucleofection.

### Constructs used in this study

EN-actin was generated as described previously (Hofmann et al. 2009). Shortly, NLS was inserted between the EYFP and actin into the plasmid pEYFP-actin (Clontech). cDNA of mouse NM1 was cloned into pCDNA3.1-mCherry using NheI and HindIII by standard methods of molecular biology.

### Indirect immunofluorescence and confocal fluorescence microscopy

U2OS cells seeded on glass coverslips were fixed with 4 % paraformaldehyde in PBS for 20 min and permeabilized with 0.1 % Triton X-100 in PBS for 10 min afterward. Non-specific labeling was further blocked with 5 % BSA in PBS for 30 min. After washes with PBS, coverslips were incubated with the respective primary antibodies diluted in PBS for 1 h at RT in a wet chamber and washed with PBST (PBS supplemented with 0.05 % Tween 20). Subsequently, coverslips were incubated with corresponding secondary antibodies for 1 h at RT in a wet chamber. After final washes in PBST, coverslips were mounted in ProLong Gold anti-fade reagent with DAPI. For detection of emerin, cells were fixed with ice-cold methanol for 5 min without additional permeabilization. Images were acquired using confocal microscope Leica TCS SP5 AOBS TANDEM with 63× (NA 1.4) immersion oil objective lens with 405, 512, 561 and 631 laser excitations, and LAS AF software.

### Antibodies

Following primary antibodies were used in this study: lamin B (Santa Cruz cat. no. sc-6217); filamin (Santa Cruz cat. no. sc-28284); alpha-actinin-4 (Abcam cat. no. ab96866); spectrin (Sigma Aldrich cat. no. S1390); paxillin (Millipore cat. no. 05-471); vinculin (Sigma Aldrich cat. no. V4505); mDia1 (BD Biosciences cat. no. P66520-050); SUN2 (Abcam cat. no. ab124916); emerin (Abcam cat. no. ab40688); Arp3 (Welch et al. 1997); cofilin (Abcam cat. no. ab11062); P-cofilin (Cell Signaling cat. no. 3313); Arp6 (Sigma Aldrich cat. no. R35554); Arp5 (Kitayama et al. 2009); Arp8 (Aoyama et al. 2008); Brg1 (Abcam cat. no. ab70558); hnRNP U (Santa Cruz, clone 3G6); H3K9Me2 (Millipore cat. no. 17-648); H3K4Me2 (Millipore cat. no. 07-030); CTD-phosphoS2 (Abcam cat. no. ab24758); RPA194 (Santa Cruz, cat. no. sc-28714); and BrdU (Sigma Aldrich, clone BU-33).

Secondary antibodies used in this study are donkey anti-rabbit IgG conjugated with Alexa Fluor 568 (A10042), goat anti-mouse IgG conjugated with Alexa Fluor 647 (A21236) and donkey anti-goat IgG cojugated with Alexa Fluor 647 (A21447) all purchased from Life Sciences.

### 5-Fluorouridine, 5-ethynyl-2′-deoxyuridine incorporation and EN-actin fluorescence measurements

U2OS cells grown on coverslips were transfected with EN-actin using Lipofectamine as described above. 48 h after the transfection, cells were incubated for 30 min or 1 h at 37 °C, 5 % CO_2_/air with 2 mM 5-fluorouridine (FU) or 5-ethynyl-2′-deoxyuridine (EdU), respectively. After this time period, cells were washed, fixed and permeabilized as mentioned above. FU incorporated into nascent transcripts was detected using anti-BrdU antibody as described above. EdU was directly labeled in a click reaction using ClickiT EdU Alexa Fluor 647 Flow Cytometry Assay kit (Life Technologies). Images were acquired as four 200-nm optical stacks of a total thickness of 2 μm using the above mentioned fluorescence confocal microscope. Total intensity of FU/EdU fluorescence in the nucleus was integrated from 3D reconstruction (maximal projection) of all four optical stacks in LAS AF, background subtracted and normalized to the nuclear area. The measurement was repeated three times, and fluorescence intensities of the cells expressing EN-actin were in each replicate normalized to the controls to prevent variations caused by antibodies dilutions, etc. Results are presented as a mean of three experiments ± standard deviation (SD) and were plotted using Prism GraphPad. *T* test was used to determine the statistical significance. Each cell imaged was manually classified according to the EN-actin expression pattern as G-actin (homogenous signal), F-actin (nuclear filaments) or control (no expression of EN-actin). Fluorescence of EN-actin was quantified in the same way.

## Results

### EN-actin forms filaments in the nucleus

**Fig. 1:**
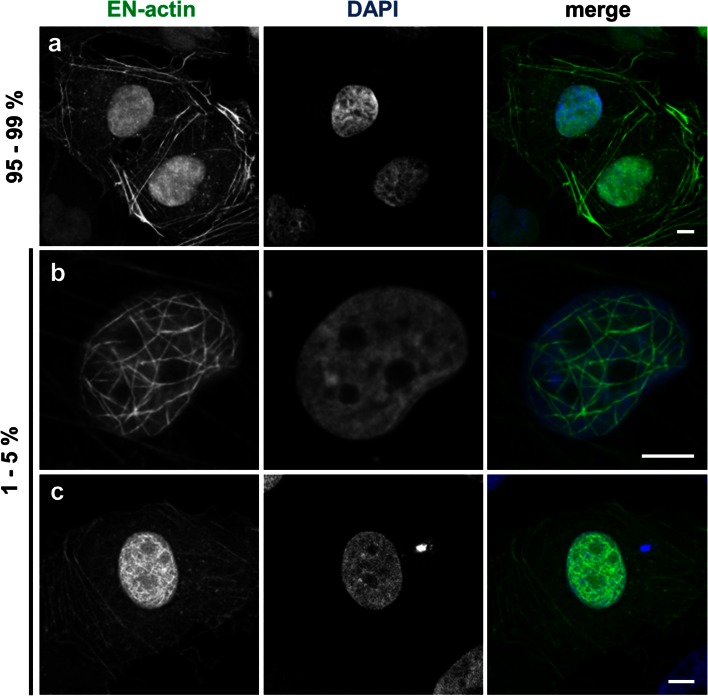
Overexpressed EN-actin forms filaments in the nucleus of U2OS cells. In vast majority of cells (95–99 %), EN-actin was imported into the nucleus, where it was homogenously dispersed throughout the nucleoplasm (**a**). Minority of cells (1–5 %) displayed EN-actin assembled into thick nuclear filaments (**b**). At the same time, EN-actin was also incorporated into cytoplasmic filaments (**a**, **c**). Single focal plane in the equatorial position (**b**) and 3D reconstructions of the entire cells (**a**, **c**) are shown. *Scale bars* 5 μm

**Fig. 2:**
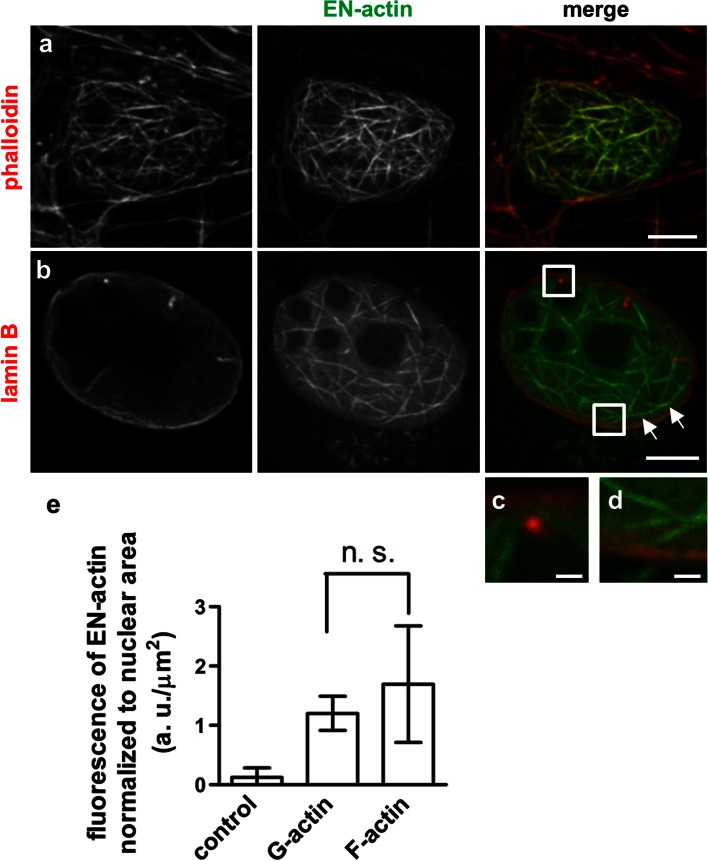
Properties of nuclear EN-actin filaments formed in U2OS cells. Nuclear EN-actin filaments are susceptible to phalloidin staining (**a**), run along the nuclear lamina (**b** *white arrows*) and occasionally join the nuclear lamina (**c**–**d**). No significant difference in the total nuclear fluorescence intensity of EN-actin normalized to the nuclear area was found between the cells forming EN-actin filaments (F-actin; **e**) and cells containing homogenously dispersed EN-actin (G-actin; **e**). As a control, cells having no expression of EN-actin but present within the same coverslip were used. Results are presented as mean ± SD of three independent experiments, whiskers indicate minimal and maximal values. In total, 30 cells for F-actin, 69 cells for G-actin and 130 control cells were analyzed (**e**). *Scale bars* 5 μm (**a**–**e**), 1.25 μm (**f**–**g**), *n. s.* *p*>0.05

We studied the behavior of exogenous β-actin in the nucleus. In order to achieve its nuclear localization, we fused β-actin with NLS and EYFP (EN-actin). It has been observed previously that the overexpression of NLS-β-actin leads to the formation of filamentous structures inside of the nucleus in various cell lines (Kokai et al. 2014). When we overexpressed EN-actin in human osteosarcoma cell line (U2OS), majority of cells (95 to 99 %) exhibited homogenously dispersed nuclear signal, apparently corresponding to the free G-actin or short actin polymers (Fig. 1a). However, in 1–5 % of cells, EN-actin assembled into filamentous structures which stretched through the whole nuclear volume with the exception of nucleoli (Fig. 1b, c). The EN-actin filaments adopt various shapes from straight long (Fig. 8h) to curved (Fig. 1b), or they form a dense meshwork (Fig. 1c). These nuclear actin filaments are phalloidin-positive structures (Fig. 2a) which in some cases run at the nuclear periphery along the nuclear lamina (Fig. 2b, white arrows), occasionally even reaching the nuclear lamina (Fig. 2c, d). The thickness of and the length of the filaments range from 50 to 100 nm and 1 to 15 μm, respectively, which corresponds to actin bundles rather than single filaments, as has been concluded previously (Kokai et al. 2014).

In parallel to its nuclear localization, EN-actin was also incorporated into canonical cytoplasmic filaments in both cells with homogenous nuclear pattern (Fig. 1a), as well as in the cells that contained nuclear EN-actin filaments (Fig. 1c). This suggests that the presence of cytoplasmic EN-actin filaments does not restrict nuclear EN-actin filaments formation, and vice versa.

Since nuclear EN-actin filaments are present only in a small fraction of cells, this raises the question which stimulus triggers their formation. One could predict that when the critical concentration of actin monomers inside a compartment is reached, the polymerization process starts. To find out whether there is a difference in the amount of EN-actin in the nucleus between the cells forming filaments and those having homogenous dispersion of EN-actin, we measured the total fluorescence intensity of EN-actin in the nuclei of those cells. Because there is a variability in size of the nuclei among the cells, we normalized total fluorescence intensity to the nuclear area after background subtraction. We found that there is no significant difference in normalized fluorescence intensity between nuclei with homogenously dispersed EN-actin (G-actin) and filaments-forming nuclei (F-actin; Fig. 2e).

In addition, we tested the impact of transfection method on the filament formation. For this purpose, we used Lipofectamine 2000 (Life Technologies), TurboFect (Thermo Scientific) and linear polyethylenimine (Polysciences) according to the manufacturers’ protocols (see Materials and methods). Even though the efficiencies of the transfections varied, the percentage of transfected cells containing nuclear actin filaments did not change significantly (data not shown).

Taken together, after overexpression of EN-actin, 1–5 % of cells contain nuclear EN-actin filaments assembled into bundles. Formation of these filaments is dependent neither on the intranuclear concentration of EN-actin nor on the transfection method.

### Formation of nuclear EN-actin filaments varies among cell types

**Fig. 3:**
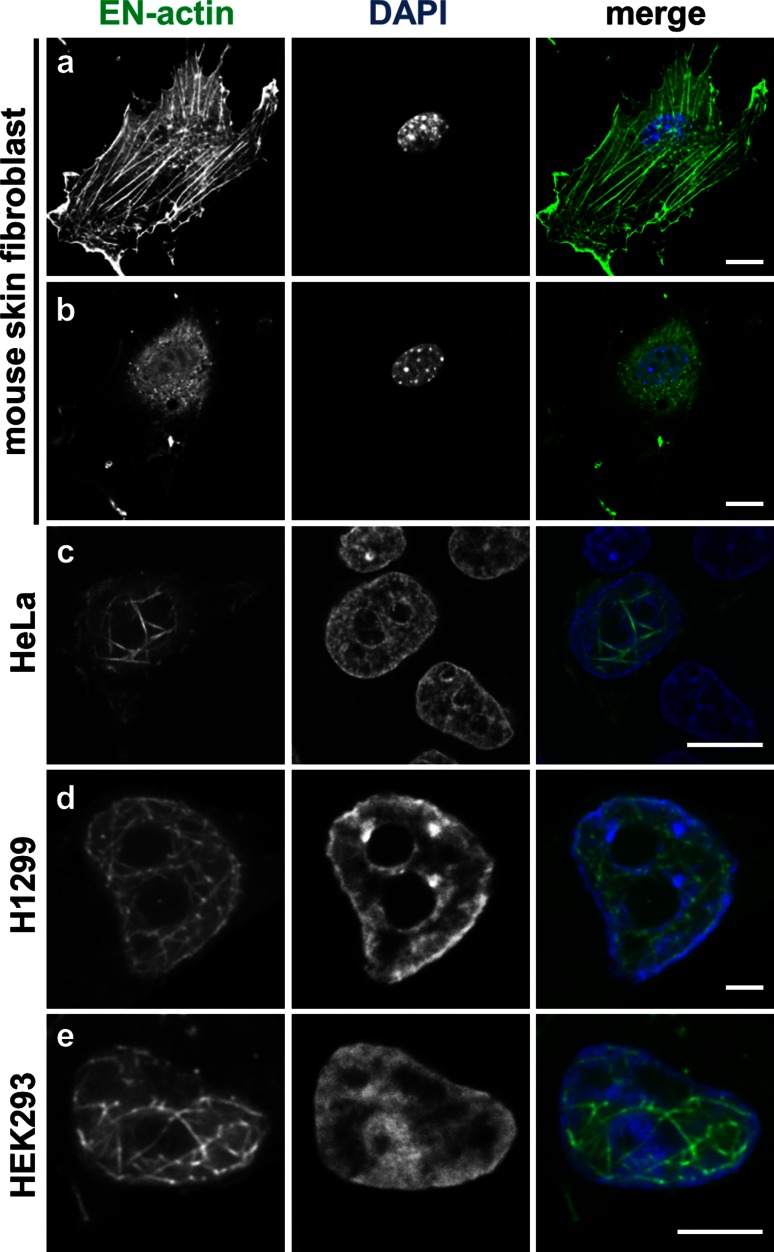
Formation of nuclear EN-actin filaments varies among cell types. In primary mouse skin fibroblasts, EN-actin (delivered by nucleofection) incorporates preferentially into cytoplasmic fibers (**a** optical section focused on the cytoplasmic fibers) and does not form filaments in the nucleus (**b** optical section of the same cell in the equatorial position). EN-actin, delivered by transfection, assembled into filaments in the nuclei of Hela (**c**), H1299 (**d**) and HEK293 (**e**) cells.* Scale bars* 10 μm (**a**–**c**), 5 μm (**d**–**e**)

We analyzed the formation of nuclear EN-actin filaments in various cell types. The pattern of overexpressed EN-actin was inspected in immortalized human embryonic kidney cell line (HEK293), human cervical carcinoma cell line (HeLa), human non-small cell lung carcinoma cell line (H1299) and primary mouse skin fibroblasts. Formation of nuclear actin filaments was noticed in all immortalized human cell lines (HEK293, HeLa, H1299; Fig. 3c–e); however, no nuclear filaments were found in primary mouse fibroblasts (Fig. 3a, b). In mouse fibroblasts, EN-actin was preferentially incorporated into cytoplasmic fibers (Fig. 3a, optical section focused to the cytoplasmic fibers), while only a small portion was imported into the nucleus, where it stayed homogenously dispersed in the monomeric form (Fig. 3b, the same cell—optical section in the equatorial position).

However, we noticed some differences between the immortalized cell lines. HEK293 cells (Fig. 3e) formed nuclear actin filaments more readily than U20S cells, reaching up to 10–20 % of cells with filaments. On the other hand, the proportion of H1299 cells forming nuclear actin filaments was only around 0.5 % (Fig. 3d). Even though we found nuclear EN-actin filaments in some H1299 cells, EN-actin was not imported into the nucleus efficiently; it rather stayed in the cytoplasmic filaments in majority of cells (not shown).

Altogether, we conclude that the ability to translocate EN-actin into the nucleus and form nuclear EN-actin filaments is cell-type specific and reflects diverse nuclear environment and/or nucleocytoplasmic transport properties.

### Cells with nuclear EN-actin filaments undergo a mitotic block

**Fig. 4:**
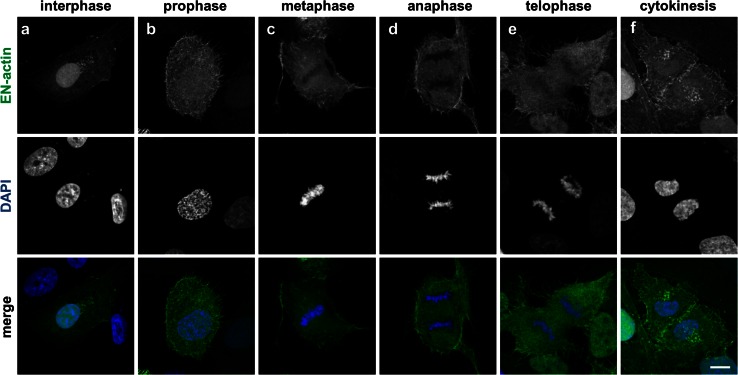
EN-actin is enriched at the plasma membrane during mitosis. Localization of overexpressed EN-actin was observed at various stages of mitosis in U2OS cells (**a**–**f**). At the onset of mitosis, EN-actin is exported from the nucleus to the plasma membrane (**b**–**e**). When the nuclear envelope re-assembles, EN-actin is imported back into the nucleus (**f**). Maximal projections of five optical sections are shown. *Scale bars* 10 μm

In order to investigate the behavior of nuclear EN-actin during cell cycle, we observed localization of homogenously dispersed EN-actin and EN-actin incorporated into the filaments at various stages of mitosis by light microscopy (Fig. 4a–f). We revealed that homogenously dispersed EN-actin is at the onset of mitosis exported from the nucleus (Fig. 4b). In later phases of mitosis, EN-actin is not associated with chromosomes; it is enriched at the plasma membrane and in plasma membrane protrusions instead (Fig. 4c–e). EN-actin is imported into the nucleus after the re-assembly of the nuclear envelope during cytokinesis (Fig. 4f).

**Fig. 5:**
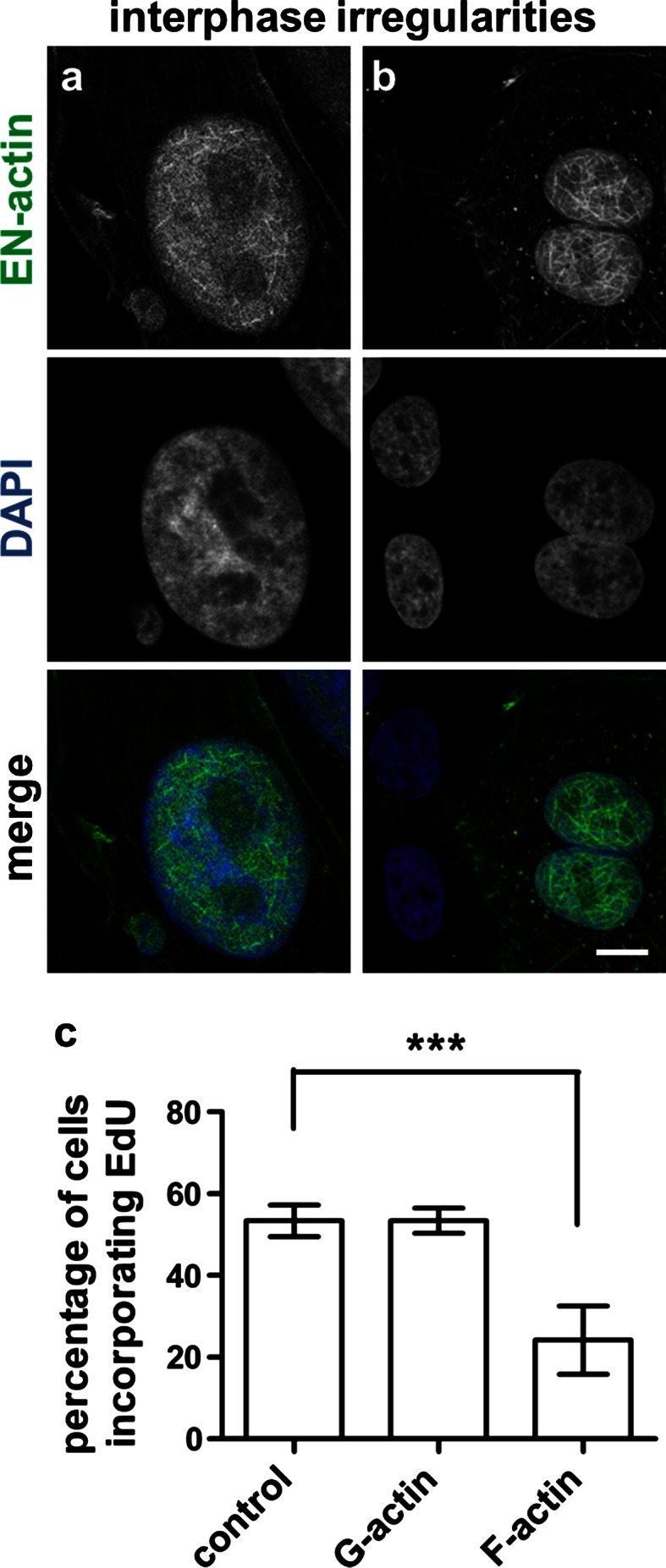
Cells with EN-actin filaments exhibit irregularities in the interphase. U2OS cells with EN-actin filaments exhibit two phenomena originated in mitosis—presence of DAPI-stainable micronuclei (**a**) and retention of both daughter nuclei within a single cell (**b**). Cell proliferation was measured by EdU incorporation. After labeling, fluorescence of EdU was measured and percentage of EdU-positive cells is shown for cells containing EN-actin filaments (F-actin, **c**), homogenous EN-actin (G-actin, **c**) and control. Results are presented as mean ± SD of three independent experiments. More than 50 cells were analyzed in each experiment (**c**). *Scale bars* 10 μm, ***p < 0.001

Interestingly, when we monitored cells by a long-term live-cell observations, we did not observe any cells containing EN-actin filaments to progress through mitosis. At the same time, other cells in the field of view which contained cytoplasmic EN-actin filaments or homogenous nuclear EN-actin divided normally (data not shown). This suggests a block in mitosis caused by the presence of EN-actin filaments in the nucleus. Indeed, when we measured proliferation rate by EdU incorporation, 53 % of the control or homogenous nuclear EN-actin containing cells incorporated EdU (Fig. 5c, control and G-actin, respectively). After the formation of nuclear EN-actin filaments, the EdU incorporation decreased by a half, to 24 % (Fig. 5c, F-actin). We furthermore noticed that many cells carrying nuclear actin filaments exhibited two types of morphological abnormalities: in the first case, additional micronuclei was formed. This micronuclei contained DAPI-stainable chromatin and also a homogenous or filamentous EN-actin (Fig. 5a). Second, some cells did not complete cytokinesis resulting in retention of both daughter nuclei within one cell (Fig. 5b). Of the binucleic cells, 90 % contained nuclear EN-actin filaments in both nuclei, while only 10 % of cells had homogenous EN-actin. The other way around, of all the nuclear EN-actin filament-containing cells, 10 % were binucleic, while only 1 % of cells with homogenous nuclear EN-actin were binucleic. In the binucleic cells, both nuclei always contained the same pattern of EN-actin—either filamentous or homogenous.

Based on the results, we propose that the presence of the EN-actin filaments in the cell nucleus may disturb progress into mitotic phase of a cell cycle. In case the cell still undergoes mitosis, irregularities in structure of daughter cells or aberrant cytokinesis appear as a consequence.

### Cofilin co-localizes with nuclear EN-actin filaments, and Arp3 is enriched in cells with EN-actin

**Fig. 6:**
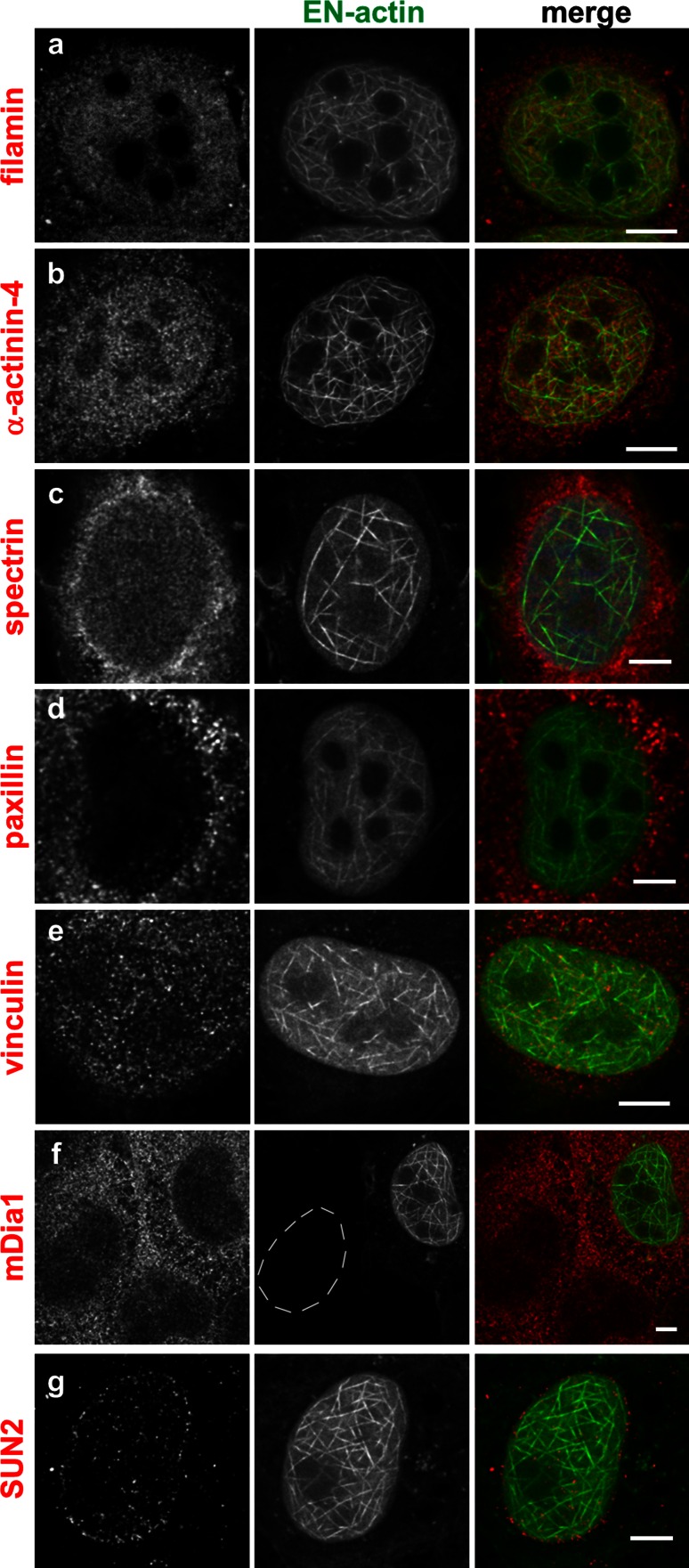
Nuclear EN-actin filaments do not co-localize with the actin-binding proteins tested. Co-localization of the nuclear EN-actin filaments with various actin-binding proteins was tested by indirect immunofluorescence microscopy in the U2OS cells (**a**–**g**). A protein was considered as co-localizing when it predominantly accumulated at the nuclear EN-filaments or was enriched in their close vicinity. Nucleus of cell with no EN-actin expression is labelled by a *dashed line*. *Scale bars* 5 μm

The initial experiment (Fig. 2e) showed that the concentration of EN-actin is not the only factor which triggers assembly of nuclear EN-actin filaments. To see whether actin-binding proteins participate in the regulation of EN-actin filaments formation in the nucleus, we observed their localization in respect of the nuclear EN-actin filaments by confocal light microscopy (Fig. 6). We considered particular protein as co-localizing when it was accumulated or enriched at the EN-actin filaments or in their close vicinity.

As we have established that EN-actin does not form individual filaments but bundles instead, we explored the localization of F-actin cross-linking proteins filamin, α-actinin and spectrin (Fig. 6a–c) which are known to localize to the nucleus (Bedolla et al. 2009; Dingova et al. 2009). None of these actin cross-linkers, however, showed preferential co-localization with nuclear EN-actin filaments; therefore, it remains unclear by which mechanism nuclear EN-actin filaments become bundled.

Next, we explored the localization of the F-actin-binding proteins paxillin and vinculin (Fig. 6d, e). These two proteins typically associate with focal adhesions, where vinculin mediates the association between integrin and F-actin and binds also paxillin (Turner et al. 1990). Despite the fact that both vinculin and paxillin were previously reported to localize in the nucleus (Dingova et al. 2009; Dong et al. 2009; Kano et al. 1996), we detected only a negligible amount of nuclear paxillin. Yet, neither of them co-localized with the nuclear filaments formed after the overexpression of EN-actin (Fig. 6d, e). Therefore, we speculate that nuclear-specific isoforms of actin-bundling proteins assist in cross-linking of EN-actin filaments.

In a recent study, Baarlink et al. (2013) showed that formation of the actin filaments in the nucleus is dependent on the presence of nuclear formins. Since we observed neither co-localization of formin mDia1 with the EN-actin filaments nor any change in pattern of mDia upon EN-actin filaments formation (Fig. 6f), we concluded that mDia1 does not assist in EN-actin polymerization.

**Fig. 7:**
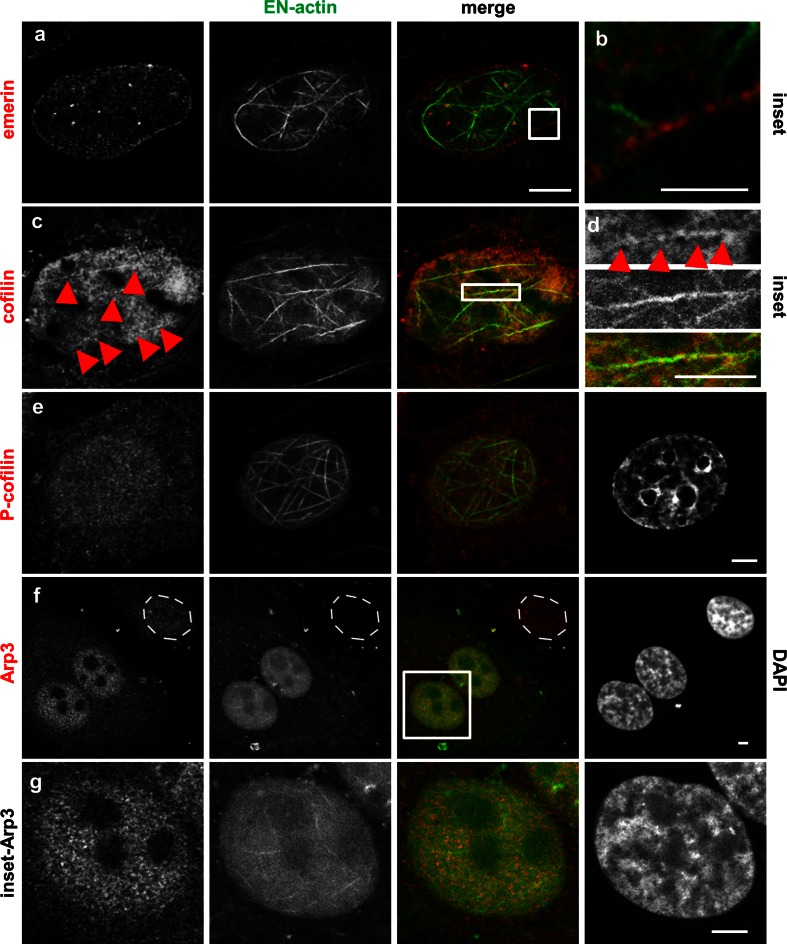
Nuclear EN-actin filaments recruit Arp3 and cofilin. Co-localization of the nuclear EN-actin filaments with various actin-binding proteins was tested by indirect immunofluorescence microscopy in the U2OS cells (**a**–**g**). EN-actin filaments occasionally come into contact with emerin (**a** and **b** *inset*). EN-actin filaments co-localize with cofilin in the nucleus (**c** and **d**, *arrowheads*). *Inset* of the EN-actin filaments (**d**). EN-actin filaments do not co-localize with P-cofilin (**e**), but recruit Arp3 into the nucleus (**f**). Nucleus of cell with no expression of EN-actin is labeled by dashed line (**f**). *Inset* of the cell with increased Arp3 levels and EN-actin filaments (**g**). *Scale bars* 2.5 μm

Our results show that nuclear EN-actin filaments join nuclear lamina occasionally (Fig. 2b–d). Therefore, we also tested their association with two other nuclear envelope-associated proteins—SUN2, a member of linker of nucleoskeleton and cytoskeleton complex (LINC; Fig. 6g); and emerin, an inner nuclear membrane protein, which binds lamin A/C (Fig. 7a, b). Of these proteins, nuclear EN-actin filaments join in some cases emerin (Fig. 7a, b) in a similar manner as lamin B (Fig. 2b–d).

Next, we investigated the localization of proteins which affect F-actin assembly. First of them, cofilin binds to the pointed end of F-actin filaments and causes their disassembly. Surprisingly, cofilin co-localized with the nuclear EN-actin filaments (Fig. 7c, arrowheads) not only at the ends, but along the entire length of the filament (Fig. 7d, arrowheads). On the contrary, phosphorylated form of cofilin (P-cofilin), which becomes incapable of F-actin binding, did not co-localize with EN-actin filaments (Fig. 7e), even though it was present in the nucleus.

Since the previous study suggested that NLS-actin filaments are branched (Kokai et al. 2014), we explored also localization of branching proteins which area able to bind to the existing filaments in order to trigger nucleation and growth of new branches of the actin filaments. We found that levels of Arp3, a member of Arp2/3 nucleation complex (Pantaloni et al. 2000), are increased upon expression of EN-actin (Fig. 7f, g). It is therefore plausible that Arp3 re-localizes to the nucleus after elevation of EN-actin to assist in the growth of new filaments.

Among the actin-binding proteins analyzed, only Arp3 and cofilin seem to be in relation with the nuclear EN-actin filaments. Such limited co-localization indicates that assembly and bundling of nuclear EN-filaments are controlled by nuclear-specific regulators or nuclear-specific isoforms of actin-associated proteins.

### Nuclear EN-actin filaments formation enhances transcription in the S-phase

**Fig. 8:**
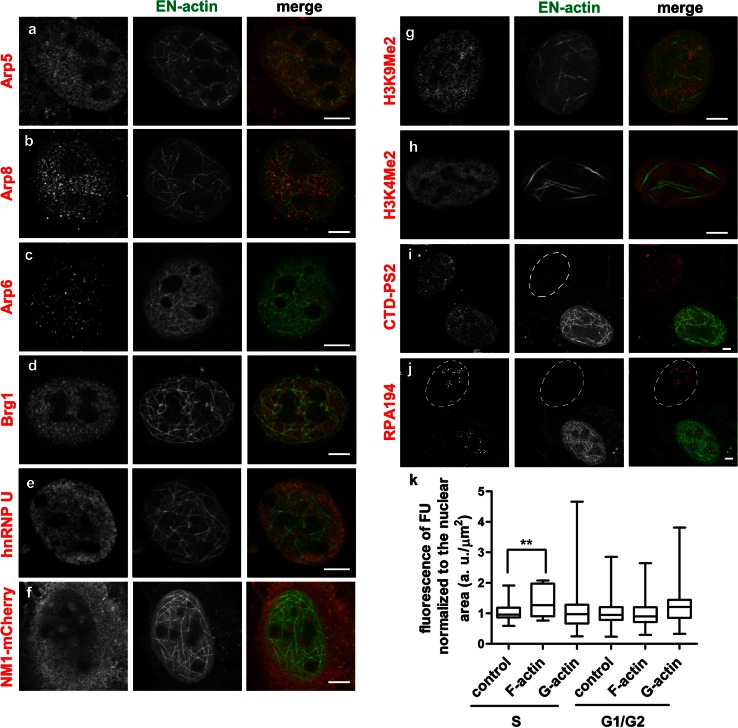
Nuclear EN-actin filaments enhance DNA transcription. Co-localization of overexpressed nuclear EN-actin and hallmarks of various nuclear functional complexes was observed in U2OS cells by indirect immunofluorescence microscopy. A protein was considered as co-localizing when it predominantly accumulated at the nuclear EN-filaments or was enriched in their close vicinity. Nuclear EN-actin filaments do not co-localize with components of chromatin remodeling complexes (**a**–**f**), but passed through both transcriptionally inactive (**g**) and active chromatin (**h**). Formation of EN-actin filaments does not affect either localization of C-terminal domain of RNA polymerase II phosporylated on serine 2 (CTD-PS2, **i**) or the catalytic subunit of RNA polymerase I (RPA194, **j**). Generation of nuclear EN-actin filaments causes increase in the overall transcription levels in the S-phase (**k**). In this experiment, transcription levels of cells containing EN-actin filaments (**k** F-actin) were compared to cells with homogenous EN-actin (**k** G-actin) and to cells with no expression of EN-actin, which resided within the same coverslips (**k** control). Nascent transcripts were labeled by FU in the U2OS cells and their amounts were then quantified by indirect immunofluorescence using anti-BrdU antibody. Total fluorescence intensity in the nucleus was normalized to the nuclear area. The experiment was repeated three times and the values in each replicate were further normalized to the control. Normalized mean values ± SD of three independent experiments are shown in the graph where whiskers represent the minimum and maximum values. More than 20 cells were analyzed in each experiment. S and G1/G2 phases of the cell cycle were analyzed separately. No significant changes (*p* < 0.05) were observed unless indicated by *asterisks*. **p = 0.01-0.001. *Scale bars* 5 μm

It is known that actin is found in chromatin remodeling complexes (Szerlong et al. 2008; Zhao et al. 1998). To test the functional involvement of the EN-actin filaments in chromatin remodeling, we performed co-localization studies with protein hallmarks of chromatin remodeling using confocal microscopy. However, no significant co-localization was observed with the actin-related proteins (Arp5, Arp8 and Arp6), brahma-related gene 1 (Brg1) or hnRNP U (Fig. 8a–e).

Numerous studies have repeatedly emphasized the importance of actin in transcription (Hofmann et al. 2004; Hu et al. 2004; Philimonenko et al. 2004). NM1 is a transcription factor, which exerts its function in cooperation with actin (Ye et al. 2008). Even though one would expect NM1, which requires oligo- or polymeric actin for its function, to be predominantly found on the EN-actin filaments, it is not the case (Fig. 8f). Overexpressed NM1-mCherry is in the nucleoplasm present in the vicinity of the EN-actin filaments, but no evidence points toward their association.

We then proceeded with the study of participation of EN-actin in transcription and observed its occurrence in transcriptionally inactive and active chromatin regions, marked by H3K9Me2 and H3K4Me2 histone modification, respectively. Nuclear EN-actin filaments did not show any preferential enrichment in either type of chromatin, neither did homogenously dispersed EN-actin (Fig. 8g, h). On the other hand, EN-actin filaments did not avoid either type of chromatin; they passed through both chromatin regions instead. Therefore, we asked whether EN-actin filaments or free EN-actin do indeed affect transcription as has been previously published (Miyamoto et al. 2011; Wu et al. 2006; Ye et al. 2008). To answer this question, we explored the presence of the catalytic subunit of RNA polymerase I (RPA194) as well as the active form of RNA polymerase II phosphorylated on serine 2 (CTD-PS2), as these would indicate active transcription. Both CTD-PS2 and RPA194 were present in the cells containing nuclear EN-actin filaments (Fig. 8i, j), and no obvious changes in their localization were noticed in comparison with non-transfected cells. In order to assess the impact of homogenous and filamentous EN-actin on transcription, we compared transcription levels of those cells with cells having no overexpression of actin (control). As it is known that transcription is inactivated during mitosis, gradually activated during G1 and its levels are maximal in S and G2 phases (Klein and Grummt 1999; Oelgeschlager 2002; White et al. 1995), we measured the transcription levels in different stages of the cell cycle based on the proliferating cell-nuclear antigen (PCNA) pattern. Nascent transcripts were labeled with fluorouridine (FU) in vivo, which was then detected by indirect immunofluorescence microscopy. Total fluorescence intensity of FU in the nucleus was quantified and normalized to the nuclear area. Transcription levels of cells expressing either homogenous EN-actin (Fig. 8k, G-actin) or EN-actin filaments (Fig. 8k, F-actin) did not significantly differ from the control (Fig. 8k, control) cells in the G1 and G2 phases of the cell cycle. However, we detected changes in transcription in the S-phase when cells forming nuclear EN-actin filaments significantly increased their transcription levels by 30 % (Fig. 8k) in comparison with control cells. On the other hand, S-phase transcription of cells having homogenous nuclear EN-actin did not significantly differ either from control cells or from the cells with EN-actin filaments.

In conclusion, nuclear EN-actin filaments do not participate in chromatin remodeling, do not preferentially associate with transcriptionally active or inactive chromatin, but their presence causes increase in general transcription levels in the S-phase in comparison with control cells.

## Discussion

The fundamental ability of actin is to form polymers. Although polymeric structures are long known to exist in the cytoplasm, their presence and form in the nucleus remains unclear.

We showed that the overexpression of EN-actin triggers formation of bundled filaments in the nucleus bearing various shapes from straight long (Figs. 7e, 8h) to curved (Fig. 7a) and dense meshwork (Figs. 1c, 6e). This observation is in agreement with a previous work by Kokai et al. (2014), which moreover proposed that some of the filaments are even branched. Even though we did not study this feature in greater detail, we support the notion that some of the filaments are indeed branched, not only crossing each other (Fig. 2b–c).

In U2OS cells, EN-actin localizes not only to the nucleus, but is also incorporated into cytoplasmic filaments (Fig. 1a and c). The incorporation of EN-actin into the cytoplasmic fibers affected neither formation of EN-actin nuclear filaments nor its nuclear translocation, which was indeed favored (Fig. 1c). The distribution of EN-actin within a cell seems to be cell-type specific, because cytoplasmic retention was not observed in rat PC12 cells (Kokai et al. 2014), whereas in primary mouse skin fibroblasts (Fig. 3a, b) EN-actin resided preferentially in the cytoplasm and did not form nuclear filaments. At the same time, EN-actin was readily imported into the nucleus of HEK293 cells (Fig. 3e). This may reflect differential requirements of actin in the nuclear processes in various cell types.

The formation of nuclear filaments after expression of exogenous EN-actin is relatively rare in U2OS cells, since only 1–5 % of cells show such phenomenon. Such a low incidence of EN-actin filament formation suggests that specific conditions are required to trigger polymerization. It is known that actin begins to polymerize when the critical concentration of free actin monomers is achieved. However, we did not observe such concentration dependency, since the expression levels of EN-actin normalized to nuclear area did not differ significantly in cells with homogenous EN-actin versus cells containing EN-actin filaments (Fig. 2e). This indicates that the amount of actin in the nucleus is not the only factor determining the filament formation, but seems to be a prerequisite. In agreement, blocking the actin export has been reported to cause actin polymerization inside of the nucleus (Dopie et al. 2012; Stuven et al. 2003).

While we observed that cells containing homogenous EN-actin progressed through mitosis (Fig. 4), during which EN-actin mimicked localization of the endogenous actin (Yang et al. 2004), the presence of nuclear EN-actin filaments decreased cell proliferation rate by a half (Fig. 5c). Moreover, we observed two abnormalities in the interphase cells which seem to originate in mitosis—formation of additional micronuclei or retention of both daughter nuclei within one cell (Fig. 5a, b). These two irregularities were previously observed by Moulding et al. (2007) as a consequence of increase in cytoplasmic F-actin assembly, which caused its mislocalization and led to delay in mitosis and defects in cytokinesis. Besides, both micronuclei formation and bridging the two daughter nuclei together are also results of improper chromosome segregation, which is caused by aberrant centromeric incidence (reviewed in Fenech et al. 2011). Because F-actin is as well required for the anchoring of mitotic spindle to the cell cortex and moreover to establish the direction of spindle movement (Woolner and Bement 2009), it is plausible that the excessive amount of overexpressed EN-actin (which may form filaments during mitosis) prevents correct spindle positioning and manifests in chromosome segregation errors. Since 90 % of the binucleic cells contained nuclear EN-actin filaments, whereas only 10 % of the cells contained homogenous nuclear EN-actin, we speculate that the effect is reinforced with increasing filamentous EN-actin levels. In conclusion, multiple aspects seem to contribute to the defects in mitosis; however, the severity is related to the amount of EN-actin which is available for polymerization into nuclear filaments.

It has been shown that the increase in cofilin expression causes arrest in G1 phase of a cell cycle by a mechanism which involves cyclin-dependent kinase inhibitor p27^Kip1^ (Tsai et al. 2009). Although we did not observe elevated levels of cofilin, we showed a co-localization between cofilin and the nuclear EN-actin filaments (Fig. 7c, d). Therefore, we speculate that cofilin might trigger the nuclear p27^Kip1^ leading to G1 arrest. In conclusion, the defective mitosis is probably a result of more than one aspect and additional experiments need to be performed to understand this issue clearly.

Numerous actin-binding proteins localize and exert their functions in the nucleus (reviewed in Castano et al. 2010). Among those tested in this study, cofilin co-localized with the nuclear EN-actin filaments (Fig. 7c, d,), whereas its phosphorylated form (P-cofilin) did not (Fig. 7e). Besides its involvement in cell cycle progression, cofilin employs multiple modes of action—upon increase in G-actin amount, cofilin maintains actin import into the nucleus (Pendleton et al. 2003) and, at the same time, regulates actin dynamics. Cofilin severs actin filaments at low actin concentrations and nucleates actin filaments at high actin concentrations (Andrianantoandro and Pollard 2006). When filaments are bundled, they become more resistant to cofilin severing (Michelot et al. 2007). Therefore, we suggest that cofilin promotes EN-actin filament formation.

We also found Arp3 upregulated upon EN-actin overexpression (Fig. 7f, g). Arp3 is a member of Arp2/3 complex, which triggers nucleation of the new or branching of the existing actin filaments (Pantaloni et al. 2000). Although we did not study branching of the EN-actin filaments, analysis of nuclear NLS-actin filaments performed by Kokai et al. (2014) revealed that the filaments are most likely branched. Hence, we propose that Arp3 might assist in EN-actin filament nucleation and branching.

Besides cofilin and Arp3, we did not identify any other actin-associated protein to co-localize with the EN-actin filaments, despite testing many potential candidates. However, as recent studies identified nuclear actin filament formation being dependent on nuclear formin (Baarlink et al. 2013), Toca-1, (Miyamoto et al. 2011), N-WASP (Wu et al. 2006) and JMY (Zuchero et al. 2009), we assume that other nuclear-specific actin-binding proteins assist in EN-actin dynamics too.

We showed here that both homogenous and filamentous forms of EN-actin are preferentially neither enriched nor excluded from the chromatin regardless of its transcriptional state (Fig. 8g, h). Noteworthy, we observed that EN-actin filaments seem to avoid only densely packed heterochromatin (Figs. 1c, 3c–e, 7g), which indeed occurs rarely in the U2OS cells as revealed by the electron microscopy (not shown). Based on the absence of co-localization between nuclear EN-actin filaments and chromatin remodeling complexes (Fig. 8a–f), we support the notion that the actin in chromatin remodeling complexes and in complex with hnRNPs is monomeric (Obrdlik et al. 2008; Percipalle et al. 2002) and nuclear EN-actin filaments do not seem to affect chromatin state.

Similarly, formation of nuclear EN-actin filaments did not affect the gross localization of active forms of RNA polymerases I and II (Fig. 8i, j), which were concentrated in discrete foci throughout the nucleolus and nucleoplasm, respectively. The pattern of transcription foci was identical to the control cells, and all the cells were transcriptionally active. After we quantified transcription levels, we found that there is an elevation in the S-phase of the cell cycle in the presence of nuclear EN-actin filaments by 30 % in comparison with control, whereas the presence of homogenous EN-actin did not affect transcription significantly (Fig. 8k). In G1 and G2 phases, the transcription levels did not differ significantly from control. It is plausible that recruitment of EN-actin filaments to the transcription complexes in the S-phase is enabled by a more permissive state of chromatin in the S-phase. This finding also points toward the possibility that polymeric state of actin is required for transcription as has been suggested previously (Miyamoto et al. 2011; Obrdlik and Percipalle 2011; Wu et al. 2006; Ye et al. 2008; Yoo et al. 2007). Up to date, numerous studies focused on the involvement of actin in transcription of SRF-regulated genes. To trigger transcription, SRF requires its cofactor MAL which is only imported into the nucleus when free of G-actin (Miralles et al. 2003; Vartiainen et al. 2007). Collectively, these studies revealed that formation of F-actin in the nucleus or in the cytoplasm depletes levels of G-actin, which cannot sequester MAL. MAL is in turn imported into the nucleus leading to upregulation of SRF-mediated transcription (Baarlink et al. 2013; Kokai et al. 2014; Stern et al. 2009; Vartiainen et al. 2007). It is therefore reasonable to speculate that the elevation of transcription upon EN-actin filaments formation that we observed occurs via exhaustion of free G-actin monomers. However, to answer this clearly, more experiments need to be performed.

To sum up, our study documents a potential for EN-actin to form filaments in the nucleus closely resembling actin filaments in the cytoplasm. Generation of nuclear EN-actin filaments recruits cofilin and Arp3 into the nucleus and affects cellular processes. Since our observations of the EN-actin polymerization, its behavior during cell cycle, co-localization with actin-binding proteins and transcriptional activity are in agreement with previous studies, we suggest that EN-actin fusion protein mimics the endogenous actin and may be used as a tool for future challenging research focusing on actin functions in the nucleus.
